# Safflower Seed Oil and Its Active Compound Acacetin Inhibit UVB-Induced Skin Photoaging

**DOI:** 10.4014/jmb.2003.03064

**Published:** 2020-06-10

**Authors:** Eun Hee Jeong, Hee Yang, Jong-Eun Kim, Ki Won Lee

**Affiliations:** 1 Biomodulation Major and Research Institute of Agriculture and Life Sciences, Department of Agricultural Biotechnology, Seoul National University, Seoul 08826, Republic of Korea; 2 Department of Food Science and Technology, Korea National University of Transportation, Jeungpyeong 27909, Republic of Korea

**Keywords:** Safflower seed oil, acacetin, UVB, skin wrinkle, anti-aging, MMP-1

## Abstract

Ultraviolet (UV) is one of the major factors harmful to skin health. Irradiation with ultraviolet accelerates the decline of skin function, causing the skin to have deep wrinkles, dryness, decreased procollagen production, and degradation of collagen. Novel materials are needed to prevent the aging of the skin by blocking the effects of UV. Safflower seed oil (*Charthamus tinctorius* L., SSO) contains significantly high levels of unsaturated fatty acids and phytochemicals. SSO has been traditionally used in China, Japan, and Korea to improve skin and hair. Our objective in this study was to determine the effect of SSO and its active compound acacetin on UVB-induced skin photoaging in HaCaT cells and human dermal fibroblasts (HDF). SSO inhibited UVB-induced matrix metalloproteinase-1 (MMP-1) at both protein and mRNA levels in HaCaT cells and HDF. MMP-1 is known to play important roles in collagen degradation and wrinkle formation. Acacetin, a type of flavonoid, is present in SSO. Similar to SSO, acacetin also inhibited UVB-induced MMP-1 protein and mRNA levels in HaCaT cells and HDF. MMP-1 mRNA is primarily regulated by the mitogen-activated kinase (MAPK) signaling pathway. Acacetin regulated the phosphorylation of JNK1/2 and c-jun, but did not inhibit the phosphorylation of ERK1/2, p38 and AKT. Taken together, these results indicate that SSO and its active compound acacetin can prevent UVB-induced MMP-1 expression, which leads to skin photoaging, and may therefore have therapeutic potential as an anti-wrinkle agent to improve skin health.

## Introduction

As the outermost organ of the body, our skin protects us against damage from physical and chemical elements [1]. The skin is in direct contact with damage-causing factors including sunlight, air pollution, and extreme temperature. Among these environmental factors, ultraviolet (UV) irradiation by chronic exposure to the Sun is the most important injury factor [2]. It accelerates the decline of skin function, causing deep wrinkles, dryness, decreased procollagen production, and degradation of collagen [3].

Solar UV radiation is divided into three categories: UVA (320-400 nm), UVB (280-320 nm), and UVC (200-280 nm) [4]. UVA and UVB reach the Earth’s surface, but UVC is effectively filtered out by the ozone layer [5]. Of UV energy that reaches Earth, UVA accounts for 90-99% while UVB accounts for the other 1-10% [4]. Among solar UV, UVB contains more energy than UVA. It is responsible for numerous biological effects on human skin, including sunburn, sun tanning, immunosuppression, and photo carcinogenesis [2]. The use of skin care products supplemented with several effective agents working through different pathways in conjunction with the use of anti-wrinkle ingredients might be an effective approach to reduce UVB-mediated photoaging [3].

Skin wrinkles are the most prominent characteristic of aging. Histologically, degradation of collagen and increased collagenase are found in wrinkled skin [6], which results from collapse of the skin dermis. Matrix metalloproteinases (MMPs) are proteinases responsible for the degradation or synthesis inhibition of the collagenous extracellular matrix (ECM) in connective tissues [2]. Collagen represents a major structural component of the ECM in dermal connective tissue [7]. When this collagen tissue collapses, the outer layer of skin sags inward and becomes wrinkled [8].

MMP-1 (collagenase), MMP-2 (gelatinase), and MMP-9 (gelatinase) are principal neutral proteinases capable of degrading native fibrillar collagens in human skin [6]. Among these MMPs, MMP-1 preferentially degrades fibrillar collagens. Repeated exposure to UVB increases MMP-1 expression in dermis and epidermis tissue [9], leading to formation of skin wrinkles as a consequence. Therefore, inhibiting expression of MMP-1, which is used as a major marker of UVB-induced skin photoaging [2], is crucial for skin collagen preservation.

**Fig. 1:**
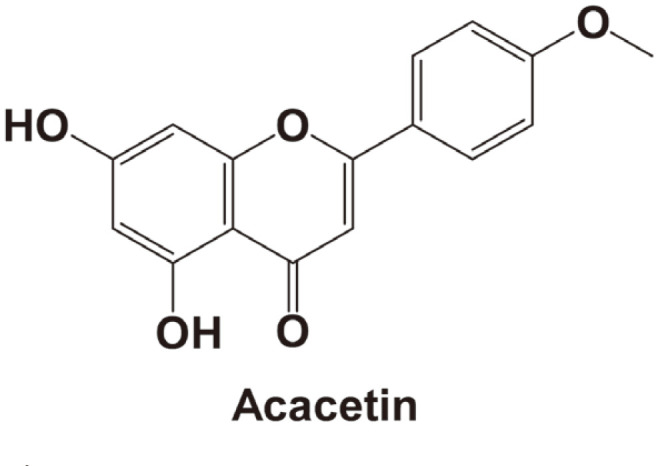
Chemical structure of Acacetin.

Safflower (*Carthamus tinctorius* L.) is widely distributed in eastern and western Asia. It has long been cultivated for oil production and coloring purposes [10]. The flower of safflower has been used in folk medicine as an analgesic, and as a crude antithrombotic, antihypertensive drug [11]. Additionally, safflower seed oil (SSO) is rich in unsaturated fatty acids such as linoleic acid and oleic acid [12]. It has long been used for industrial purposes as drying oil, as well as in meal, birdseed, cosmetics, and for producing varnish [13-15]. Recent studies have revealed that safflower seed oil with high linoleic content can improve lipid metabolism and reduce blood cholesterol level [16, 17]. It also protects against bone fracture and loss [18, 19]. Acacetin (5,7-dihydroxy-4′-methoxyflavone, Fig. 1) is a flavonoid isolated from safflower seeds, plants, and leaves [20]. Acacetin possesses anti-peroxidative, anti-inflammatory, anti-plasmodial, and anti-proliferative activities [21, 22]. Although a broad range of biological and pharmacological activities of safflower seed oil and its active phenolic compound acacetin have been documented, its anti-wrinkle or anti-photoaging effects have not yet been reported [23, 24].

Therefore, the objective of this study was to determine the effect of SSO and acacetin on UVB-induced skin photoaging in HaCaT cells and human dermal fibroblasts (HDF). We found that SSO and acacetin possessed significant inhibitory effects on UVB-induced MMP-1 expression in HaCaT cells (immortalized human keratinocyte) and primary cultured human dermal fibroblasts via down-regulation of JNK1/2 and c-Jun phosphorylation. These results provide evidence of the therapeutic potential of SSO and acacetin as anti-wrinkle agents.

## Materials and Methods

### Chemicals

Safflower seed oil was obtained from the Kerfoot Group (USA). Acacetin was purchased from Sigma-Aldrich (USA). Dulbecco’s modified Eagle’s medium (DMEM) was purchased from HyClone (USA). Fetal bovine serum (FBS) and β-actin antibody were bought from Sigma-Aldrich. MMP-1 antibody was purchased from R&D Systems Inc. (USA). Antibodies against phosphorylated extracellular signal-regulated kinase 1/2 (ERK1/2)(Thr^202^/Tyr^204^), total ERK1/2, total c-Jun N-terminal kinase 1/2 (JNK1/2), phosphorylated-p38 (Thr^180^/Tyr^182^), total p38 phosphorylated-c-jun (Ser73), total c-jun, phosphorylated-Akt (Ser473), and total Akt were purchased from Santa Cruz Biotechnology (USA). Other antibodies were obtained from Cell Signaling Biotechnology (USA). In addition, 3-[4,5-dimethylatiazol-2-yl]-2,5 diphenyltetrazolium bromide (MTT) powder was purchased from USB Co. (USA) while penicillin/streptomycin was purchased from Invitrogen (USA). Protein assay kits were obtained from Bio-Rad Laboratories (USA) and 4-(4-Aminophenyl)-1H-indazol-3-ylamine (3-aminoindazole compound) was obtained from Merck Millipore (UK).

### Cell Culture

Primary human dermal fibroblasts were isolated from the outgrowth of foreskin obtained from healthy volunteers aged 7 to 30 years old. They were provided by Dr. Chung JH at Seoul National University Hospital (Korea). This study was approved by the Institutional Review Board at Seoul National University Hospital and Seoul National University. Human dermal fibroblasts were cultured in DMEM supplemented with 10% (v/v) FBS and 1% (v/v) penicillin/streptomycin at 37 °C and 5% CO_2_. Immortalized human keratinocyte HaCaT cells (N.E. Fusenig, Germany) were cultured under the same condition described above for human dermal fibroblasts.

### UVB Irradiation

UVB irradiation was performed in serum-free media. Spectral peak of UVB source (Bio-Link Crosslinker, VilberLourmat, Cedex 1, France) was set at 312 nm. Primary human dermal fibroblasts were exposed to UVB at a dose of 0.02 J/cm^2^ while HaCaT cells were exposed to UVB at a dose of 0.01 J/cm^2^.

### Cell Viability

Cell viability was determined using MTT assay. Briefly, HDF cells were cultured in 96-well plates at a density of 2×103 cells/well and incubated in DMEM-10% FBS containing penicillin/streptomycin at 37°C in a 5% CO_2_ atmosphere. HaCaT cells were cultured in 96-well plates at a density of 10 × 10^5^ cells/well and incubated at the same condition as described above for HDF. Cells were starved in serum-free DMEM for 24 h. After cells and SSO or acacetin were incubated at 37°C for 22 h, they were treated with MTT solution and incubated for 2 h. The medium was removed and formazan crystals were dissolved by adding dimethyl sulfoxide (DMSO). Absorbance at 570 nm was then measured using a microplate reader (Molecular Devices, USA). Viabilities of cells treated with SSO or acacetin were then compared to those of untreated cells (100%).

### Western Blot Analysis

HDFs and HaCaT cells were cultured for 48 h and the cells were incubated in serum-free DMEM for 24 h. After that, the cells were treated with or without various concentrations of acacetein, followed by UVB irradiation. The media were harvested on ice, and then centrifuged at 18,620 g for 10 min. Cells were harvested and disrupted with lysis buffer (Cell Signaling). The protein concentration was measured using a protein assay reagent kit as instructed by the manufacturer. The proteins were separated electrophoretically using a 10% SDS-polyacrylamide gel and transferred onto an Immobilon P membrane (MERCK Millipore, Germany). The membrane was blocked in 5% fat-free milk for 1 h, and then incubated with the specific primary antibody at 4°C overnight. Protein bands were visualized using a chemiluminescence detection kit (GE Healthcare, UK) after hybridization with the HRP-conjugated secondary antibody (Life Technologies, USA). Western blot data were quantified using the program ImageJ (National Institutes of Health, USA).

### Gelatin Zymography

Gelatin zymography was performed on 12% polyacrylamide gel in the presence of gelatin (0.1% w/v) as a substrate for MMP-2. Protein samples were mixed with loading buffer [10% SDS, 25% glycerol, 0.25 M Tris (pH 6.8) and 0.1% Bromophenol blue] and then run on 12% SDS-PAGE gel without denaturation. Afterward, gel was washed with renaturating buffer (Life Technologies) for 1 h at room temperature and incubated with developing buffer (Life Technologies) at 37°C for 24 h. After enzyme reaction, the gel was stained with 0.5% Coomassie brilliant blue in 10% acetic acid.

### Real-Time Reverse Transcriptase PCR

Human dermal fibroblasts and HaCaT cells were treated with SSO and acacetin for 24 h and harvested in RNAiso Plus (Takara Bio, Inc., Japan). RNA was quantified using a NanoDrop ND-2000 spectrophotometer (Thermo Fisher Scientific, USA). After reverse transcription with oligo-dT primers using a PrimeScript 1st strand cDNA Synthesis Kit (Takara Bio Inc.), real-time quantitative RT-PCR was performed using IQ SYBR (Bio-Rad Laboratories) and 2 μl of cDNA in triplicates. Glyceraldehyde 3-phosphate dehydrogenase (GAPDH) was used as an internal control. Before PCR amplification, primers were denatured at 95°C for 3 min. Amplification included 44 cycles of 95°C for 10 sec, 60°C for 30 sec, and 72°C for 30 sec. PCR was performed on a CFX Connect Real-Time PCR Detection System (Bio-Rad Laboratories, USA). cDNA was probed with the following primer sets: MMP-1 forward (5’-CCC CAA AAG CGT GTG ACA GTA-3’) and MMP-1 reverse (5’-GGT AGA AGG GAT TTG TGC G-3’); GAPDH forward (5’-GAG TCA ACG GAT TTG GTC GT-3’) and GAPDH reverse (5’-TTG ATT TTG GAG GGA TCT CG-3’).

### Statistical Analysis

All experiments were repeated 3 times, and all data were analyzed using SPSS Statistics software v.23.0 (IBM Co., USA). The difference between the control group and the solar UV-irradiated control group was evaluated by Student’s t-test. The differences between the sun UV exposure groups were compared using Duncan’s multi-range test and one way ANOVA. A probability value of less than 0.05 was used as the criterion for statistical significance.

## Results

### Safflower Seed Oil Inhibits UVB-Induced MMP-1 Protein Expression in HaCaT Cells and Human Dermal Fibroblasts

**Fig. 2:**
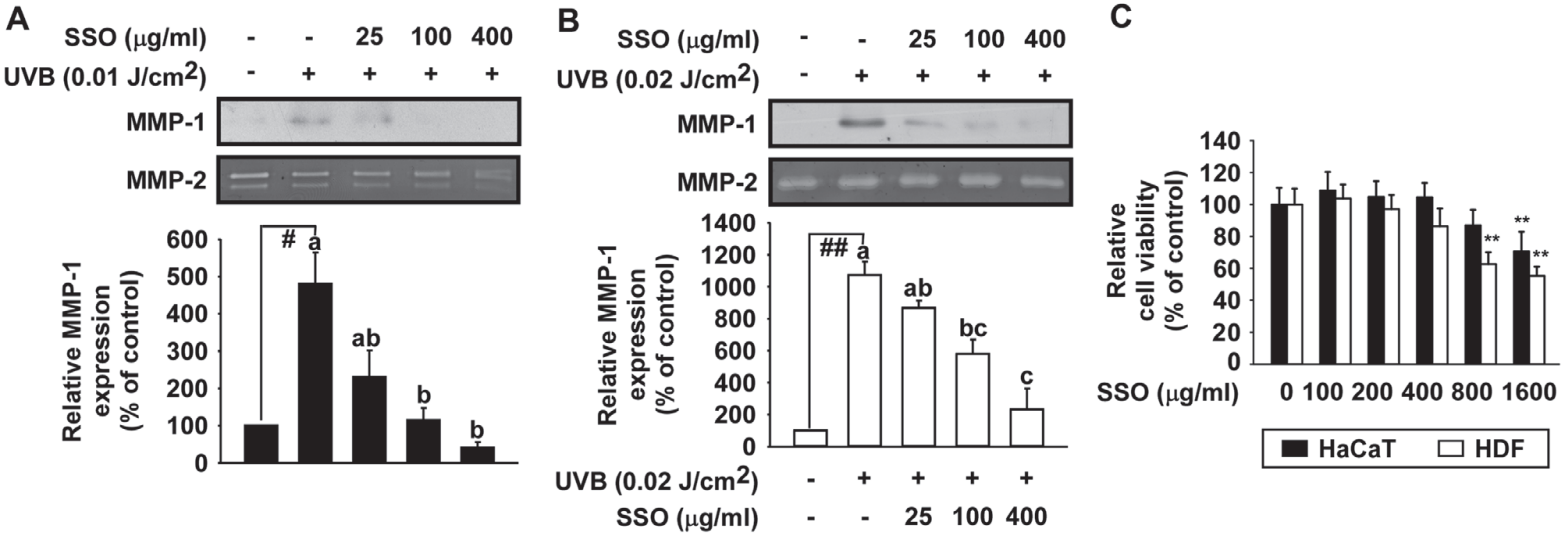
Effect of safflower seed oil (SSO) on UVB-induced MMP-1 protein expression in HaCaT cells and human dermal fibroblasts. (**A**) HaCaT cells were pretreated with SSO at indicated concentrations for 1 h and further treated with 0.01 J/cm^2^ UVB for 48 h at 37°C. (**B**) Human skin fibroblasts (HDF) were pretreated with SSO at indicated concentrations for 1 h and further treated with 0.02 J/cm^2^ UVB for 48 h at 37°C. MMP-1 protein expression data of HDF were quantified using NIH ImageJ software. Data (*n* = 3) are presented as mean ± SD. (C) Cell viability of SSO was determined using MTT assay as described in Materials and Methods. Data (*n* = 5) shown are presented as means ± SD. #*p* < 0.05 and ##*p* < 0.01 relative to control cells. *p < 0.05 and **p < 0.01 relative to UVB-induced cells. The experiment was performed in triplicate and bars marked with different letters (a–c) are significantly different (*p* < 0.05, ##*p* < 0.01), relative to the control cells.

To investigate the anti-wrinkle effect of SSO, its inhibitory effects on UVB-induced MMP-1 protein expression in HaCaT cells and HDF were examined by western blot analysis. SSO significantly suppressed UVB-induced MMP-1 expression levels in HaCaT cells (Fig. 2A) and HDF (Fig. 2B). At the concentrations (25, 100, and 400 μg/ml) used in this study, SSO did not show cytotoxicity (Fig. 2C). The intensity of MMP-1 protein expression was quantified using NIH Image software. The results showed that SSO significantly inhibited UVB-induced MMP-1 protein expression in both HaCaT and HDF (Fig. 2).

### Safflower Seed Oil Inhibits UVB-Induced MMP-1 mRNA Expression in HaCaT and Human Dermal Fibroblasts

**Fig. 3:**
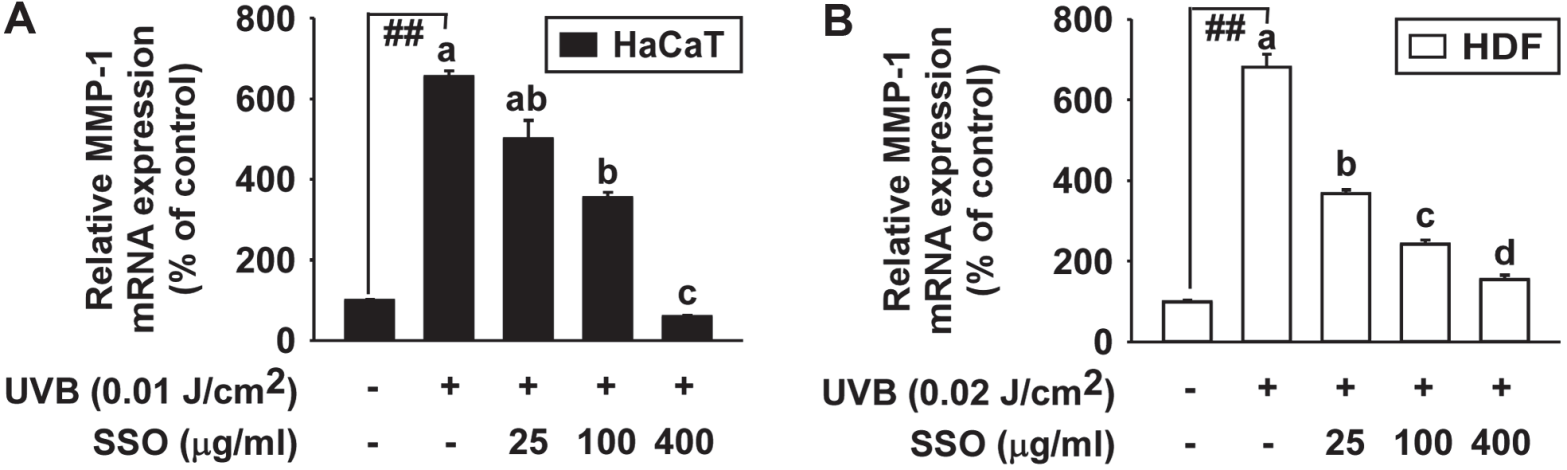
Effect of SSO on UVB-induced MMP-1 gene transcription in HaCaT cells and human dermal fibroblasts. (**A** and **B**) MMP-1 mRNA levels of SSO were analyzed by real-time quantitative PCR. Cells were pretreated with SSO at indicated concentrations for 1h and then further treated with 0.01 J/cm^2^ UVB for HaCaT cells (**A**) and 0.02 J/cm^2^ UVB for HDF (**B**) at 37°C for 48 h. Data (*n* = 3) represent the mean ± SD. ##*p* < 0.01 relative to control cells. The experiment was performed in triplicate and bars marked with different letters (a–c) are significantly different (*p* < 0.05, ##*p* < 0.01), relative to the control cells.

To investigate the inhibitory effects of SSO on UVB-induced MMP-1 mRNA expression, real-time PCR was performed. The results revealed that SSO significantly suppressed UVB-induced MMP-1 mRNA expression in HaCaT (Fig. 3A) and HDF (Fig. 3B) in a dose-dependent manner.

### Acacetin Effectively Attenuates UVB-Induced MMP-1 Protein Expression in HaCaT Cells and Human Dermal Fibroblasts

**Fig. 4:**
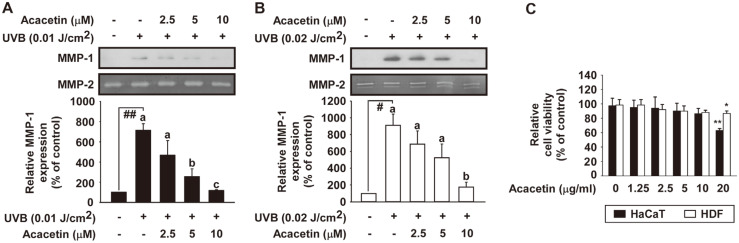
Effect of acacetin on UVB-induced MMP-1 protein expression in HaCaT cells and human dermal fibroblasts. (**A**) HaCaT cells were pretreated with acacetin at indicated concentrations for 1 h and then further treated with 0.01 J/cm^2^ UVB for 48 h at 37°C. (C) MMP-1 protein expression data of HaCaT cells were quantified using NIH ImageJ software. Data (*n* = 3) represent the mean ± SD. (**B**) HDF were pretreated with acacetin at indicated concentrations for 1 h and then further treated with 0.02 J/cm^2^ UVB for 48 h at 37°C. (E) MMP-1 protein expression data of HDF were quantified using the NIH ImageJ software. Data (*n* = 3) represent the mean ± SD. (C) Cell viability of acacetin was measured using MTT assay. Cells were starved in serum-free DMEM for 24 h. Cells and acacetin were then incubated at 37°C for 22 h followed by treatment with MTT solution for 2 h. Data (*n* = 5) represent the mean ± SD. The experiment was performed in triplicate and bars marked with different letters (a–c) are significantly different (*p* < 0.05, ##*p* < 0.01), relative to the control cells.

Acacetin is an abundant substance present in SSO [20]. To verify the anti-aging effect of acacetin, its inhibitory effects on UVB-induced MMP-1 protein expression in HaCaT cells and HDF were examined. Acacetin significantly attenuated UVB-induced MMP-1 protein expression in HaCaT (Fig. 4A) and HDF (Fig. 4B). At concentration as high as 10 μM, acacetin did not show any cytotoxicity to either cell (Fig. 4C).

### Acacetin Inhibits UVB-Induced MMP-1 mRNA Expression in HaCaT and Human Dermal Fibroblasts

**Fig. 5:**
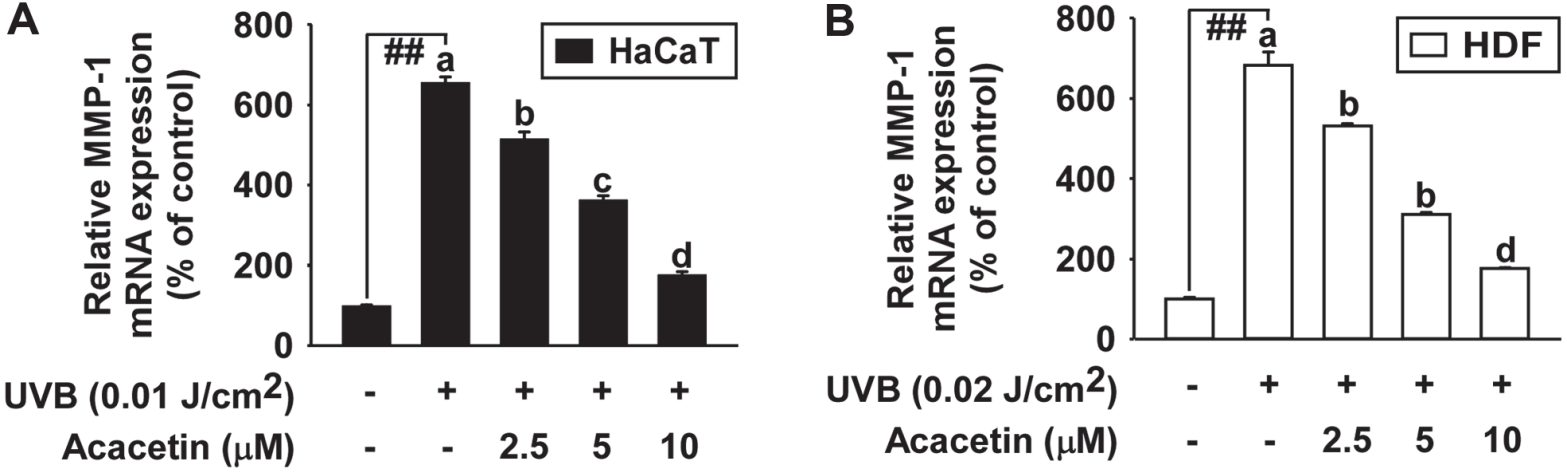
Effect of acacetin on UVB-induced MMP-1 gene transcription in HaCaT cells and human dermal fibroblasts. (**A** and **B**) MMP-1 mRNA levels of SSO were examined by real-time quantitative PCR. Cells were pretreated with SSO at indicated concentrations for 1 h and then further treated with 0.01 J/cm^2^ UVB for HaCaT cells (**A**) and 0.02 J/cm^2^ UVB for HDF (**B**) at 37°C for 48 h. Data (*n* = 3) represent the mean ± SD. The experiment was performed in triplicate and bars marked with different letters (a–c) are significantly different (*p* < 0.05, ##*p* < 0.01), relative to the control cells.

To investigate the inhibitory effect of acacetin on UVB-induced MMP-1 mRNA expression, real-time PCR was performed. The results showed that acacetin significantly inhibited UVB-induced MMP-1 mRNA expression in HaCaT (Fig. 5A) and HDF (Fig. 5B) in a dose-dependent manner.

### Acacetin Inhibits UVB-Induced Phosphorylation of JNK1/2 Signaling

**Fig. 6:**
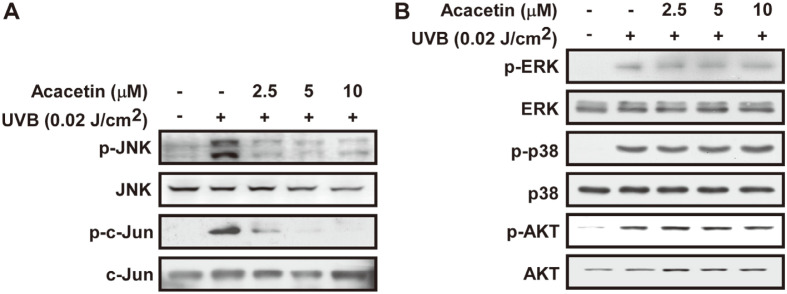
Effect of SSO and acacetin on UVB-induced phosphorylation of MAPK signaling. (**A** and **B**) After acacetin treatment and UVB irradiation (0.02 J/cm^2^), the HaCaT cells were lysed as described in the Materials and Methods. Phosphorylated and total forms of indicated proteins were determined by western blotting as described in the Materials and Methods. Total forms of each protein were used as a loading control.

It has been reported that MAPK and AKT signaling pathways play an important role in regulating MMP-1 expression [25]. Acacetin inhibits UVB-induced phosphorylation of JNK1/2 and c-jun in HaCaT cells (Fig.6A). However, acacetin could not inhibit UVB-induced phosphorylation of ERK1/2, p38 and AKT in HaCaT cells (Fig 6B). These results suggested that inhibition of JNK1/2 by acacetin leads to the suppression of MMP-1 expression.

## Discussion

Recent studies have indicated that sun exposure is a major environmental factor that aggravates skin aging. Skin aging is a process of senescence. It is commonly associated with wrinkling, sagging, and laxity [6]. Skin aging can be divided into two types: extrinsic and intrinsic aging. Extrinsic aging is generally considered as photoaging since it is caused by intense and chronic UV exposure. Intrinsic aging is characterized by smooth, dry, pale, and finely wrinkled skin. However, extrinsic photoaging is characterized by severe wrinkling and pigmental changes [6]. In recent years, many compounds have gained attention as anti-aging agents for cosmetics. For example, it has been reported that retinoic acid derived from vitamin A has the most protective effect against UV-induced skin wrinkle in human, among various agents used to treat skin wrinkles [2]. However, retinoic acid is easily degraded by light. In addition, it shows phototoxicity potential. Topical retinoic acid therapy can induce inflammation such as retinoid dermatitis. These concerns restrict the usage of retinoic acid as a cosmetic ingredient [26]. Therefore, alternative ingredients of anti-aging agents need to be developed.

UV generated from the sun is mostly absorbed by the ozone layer. However, considerable amounts of UV reach the ground [27]. UV plays a positive role in generating vitamin D. However, it also causes aging by causing generation of reactive oxygen or DNA mutation in skin cells [28]. The skin layers that UV reaches consist of the outermost epidermis and the inner dermis. In skin aging, enzymes derived from both epidermal cells and dermal cells by ultraviolet rays can collapse connective proteins such as collagen and elastin, leading to wrinkles [29]. Although both epidermis and dermis cells are skin cells, their characteristics are different. Therefore, both cells must be used to evaluate a material’s effect [6]. In this study, we examined the effect of SSO and acacetin on skin wrinkles using both HaCaT epidermis cells and HDF dermis cells.

Even though many cosmetic materials work well, they cannot be used unless they are formulated. It is important to add fragrance and color [30]. If the material has a good history or image, products containing it can reach consumers much more easily [31]. Safflower has been cultivated for a long time to obtain vegetable oil from its seeds [32]. It is widely grown in eastern and western Asia [18, 19]. Historically, it has been used as a cosmetic material to make skin smooth. It is also used as a red coloring material for makeup [13]. SSO is rich in unsaturated fatty acids and contains many fat-soluble ingredients [12]. In addition, SSO has excellent skin affinity and absorption ability with excellent oxidation stability. These advantages are important when making cosmetic formulations [33]. SSO also has a variety of effects that can improve skin health [34]. It has been found that safflower seed oil has skin-protecting, -moisturizing, and -whitening effects through inhibiting tyrosinase activity [11]. In this study, we found that SSO inhibited the expression of UV-induced MMP-1, indicating that it could be used as an anti-aging agent. SSO has excellent advantages if it is used in cosmetics.

Acacetin (5,7-dihydroxy-4′-methoxyflavone, Fig. 1) is a major flavonoid isolated from safflower seed oil. It has inhibitory effects on melanogenesis [35]. Considering that acacetin is an active component of SSO, we also examined its effect on UV-induced skin wrinkles in this study. It has been reported that acacetin can improve inflammation and inhibit skin tumor induced by phorbol ester [36]. Our previous study showed that acacetin can inhibit skin cancer [37]. Epidermal growth factor (EGF) directly activates EGFR and activates downstream signals such as PI3K. On the other hand, because UV activates signal transduction in various ways, sometimes inhibitors do not work well [38, 39]. In our previous study, acacetin inhibited EGF-induced AKT phosphorylation, but this study did not inhibit UVB-induced AKT phosphorylation. However, no studies have reported its effect on UVB-induced skin wrinkles. However, through this study we found that acacetin significantly inhibited UVB-induced MMP-1 expression in both HaCaT and HDF cells. SSO may have many substances that can improve skin aging, as demonstrated by the inhibitory activity against UVB-induced MMP-1 expression exhibited by acacetin.

MAPK and AKT pathways are activated by UV [40]. These pathways play important roles in the signal transduction system to increase MMP-1 expression. Many anti-aging agents can regulate MMP-1 through these signaling pathways [3]. We examined the UVB-induced phosphorylation of MAPK and AKT. Acacetin inhibited JNK1/2 and its downstream signal, c-jun. However, acacetin could not inhibit UVB-induced phosphorylation of ERK1/2, p38 and AKT. In our previous study, acacetin inhibited AKT by directly inhibiting PI3K [37]. It was a signaling system induced by EGF. However, in this study, we used UVB, which is different from activating receptors directly like EGF and indirectly by signaling systems like UVB. Acacetin could not inhibit UVB-induced phosphorylation of AKT.

In summary, SSO and its active compound acacetin can regulate MMP-1 expression. Although further study is needed to identify the molecular target of SSO and acacetin, our results suggest that SSO and acacetin have excellent potential as alternative anti-wrinkle ingredients.
